# In Vitro Antioxidant and Antimicrobial Properties of Flower, Leaf, and Stem Extracts of Korean Mint

**DOI:** 10.3390/antiox8030075

**Published:** 2019-03-26

**Authors:** Chang Ha Park, Hyeon Ji Yeo, Thanislas Bastin Baskar, Ye Eun Park, Jong Seok Park, Sook Young Lee, Sang Un Park

**Affiliations:** 1 Department of Crop Science, Chungnam National University, 99 Daehak-Ro, Yuseong-gu, Daejeon 34134, Korea; parkch804@gmail.com (C.H.P.); guswl7627@gmail.com (H.J.Y.); bastinbt20@yahoo.com (T.B.B.); yeney1996@cnu.ac.kr (Y.E.P.); 2 Department of Horticultural Science, Chungnam National University, 99 Daehak-ro, Yuseong-gu, Daejeon 34134, Korea; jongseok@cnu.ac.kr; 3 Marine Bio Research Center, Chosun University, 61-220 Myeongsasimni, Sinji-myeon, Wando-gun, Jeollanamdo 59146, Korea

**Keywords:** Korean mint, antioxidant activity, antibacterial activity, phenolics

## Abstract

Traditionally, *Agastache rugosa* (Korean mint) has been widely used to treat various infectious diseases. The aims of this study were to: (i) determine the phenylpropanoid content of the plant using high-performance liquid chromatography; (ii) undertake total anthocyanin, flavonoid, and phenolic assays; (iii) and evaluate the antioxidant and antibacterial properties of the methanol extracts from the stem, leaves, and flowers of Korean mint. The total anthocyanin, flavonoid, and phenolic content assays showed that the flowers had higher phenolic levels than the stem and leaves. The reducing power, the 2,2-diphenyl-1-picrylhydrazyl superoxide radical scavenging abilities, and the hydrogen peroxide radical scavenging activities were also evaluated so that the antioxidant activities of the extracts from the different plant parts could be evaluated. The flower extracts revealed higher antioxidant properties than the other parts. The antibacterial properties of the methanol extracts from *A*. *rugosa* were analyzed by the disc diffusion method, and the flower extracts had higher antibacterial activities against the six bacterial strains used in the study than the other parts. This study provides information on the synergistic antioxidant and antibacterial properties of phenolics derived from the different parts of Korean mint.

## 1. Introduction

*Agastache rugosa*, which is also called Korean mint, belongs to the Lamiaceae family, and is a traditional medicinal and ornamental plant. It is mainly distributed in East Asia, and has been commercially cultivated and used as a spice or to prepare perfumes [1]. In particular, *A*. *rugosa* has been traditionally used as a herbal remedy for the treatment of anorexia, vomiting [2], cholera, and miasma [3,4,5,6]. Previous studies have reported that *A*. *rugosa* has various pharmacological and physiological properties, including anti-cancer [7], antibacterial [8], anti-fungal [9], and antiviral activities [10]. Bioassays of *A*. *rugosa* extracts have revealed a range of pharmacological and biological actions, including anti-fungal and anti-HIV properties, and it also inhibits *Ha emophilus influenza* adhesion to human cells [11]. Moreover, Oh et al. (2006) reported that ethyl acetate extracts of Korean mint have antibacterial, antioxidant, anti-mutagenic, and anti-cancer properties [12]. These activities are attributable to the various bioactive compounds that are present in *A*. *rugosa*. Previous studies have shown that *A*. *rugosa* contains many secondary metabolites, including phenylpropanoids, carotenoids, and terpenoids. Among these metabolites, the phenolic compounds from *A*. *rugosa* have been reported to have some biological activities; namely, the compounds are anti-complementary, anti-viral, anti-fungal, anti-inflammatory, and anti-atherogenic. Furthermore, Wilson et al. (1992) reported that Korean mint contains large concentrations of tilianin, which has been demonstrated to possess anti-atherogenic and anti-inflammatory properties [13].

Plant secondary metabolites are molecules that are not essential to plant survival, but have important plant development, growth, reproduction, and protection roles [14]. In particular, phenolics, which are widely distributed in higher plant species, possess biological properties associated with plant defense against biological and physical stresses [15,16]. Furthermore, the intake of edible plants containing many phenolic compounds is beneficial to human health due to their biological activities, as well as their anti-allergic, anti-cancer, anti-microbial, and antioxidant properties [17].

Reactive oxygen species (ROS) is a collective term that includes both oxygen and non-radicals that are generated during normal metabolic processes [18]. A serious imbalance between the production and scavenging of ROS induces oxidative stress, leading to various diseases, such as allergies, cancer, cardiac and vessel injuries, and infectious and neurodegenerative diseases [19]. Many scientific studies have reported that antioxidants play an important role in reducing the pathological conditions caused by the effects of free radicals, because the antioxidant agents are stable enough to scavenge or deactivate the oxidants [20]. Plants are considered to be a good source of natural antioxidants because they contain a variety of secondary metabolites that have antioxidant capacities [21,22]. Therefore, the dietary intake of plant-derived antioxidants should be increased to prevent oxidative stress and reduce the need to take additional medication [23].

This study determined the phenylpropanoid contents in *A*. *rugosa* by High Performance Liquid Chromatography (HPLC), and total phenolic, flavonoid, and anthocyanin assays were used to investigate the antioxidant and antibacterial activities of extracts from the stems, flowers, and leaves.

## 2. Materials and Methods 

### 2.1. Plant Material

Two-month-old healthy and fresh leaves, flowers, and stems of Korean mint were harvested from the greenhouse at Chungnam National University, Daejeon, Korea. The cultivated plant materials were authenticated by a botanist in the department, and the plants were deposited under the voucher number SUP-16-0020. The harvested flowers, leaves, roots, and stems were washed with distilled water and then frozen in liquid nitrogen. Afterwards, these samples were freeze-dried in a freeze dryer (FD8512, Ilshin Lab Co. Ltd., Yangju, Korea) for 72 h, operating at −60 °C and 1.33 Pa. The dried organs were powdered using a pestle and mortar for further studies.

### 2.2. Total Phenolic Content

Fine powders (100 mg) made from each plant part were extracted with 3 mL of methanol and sonicated for 1 h. The extract was then centrifuged at 20,929× *g* and filtered through a 0.45-µm polytetrafluoroethylene (PTFE) hydrophilic syringe filter into a vial. The extracts were used for further experiments to determine the total phenolic, flavonoid, and anthocyanin contents. The Folin–Ciocalteu method was used to quantify the total phenolic content [24]. Exactly 100 μL of crude extract was mixed with 3 mL of distilled water, and then 500 µL of 2N Folin and Ciocalteu’s phenol reagent (Sigma-Aldrich Co., Yongin, Korea) was added. After incubation for 3 min at 28 °C, 2 mL of sodium carbonate (20%, *w/v*) was added to the mixture, which was then incubated for 1 h in the dark. A UV-1800 spectrophotometer (Shimadzu Corp., Kyoto, Japan) was used to measure the absorbance of each sample at 760 nm. A calibration curve equivalent (standard curve equation: *y* = 0.002*x* − 0.0226, *r*^2^ = 0.9997) was prepared using different gallic acid (Sigma-Aldrich Co., Yongin, Korea) solution concentrations, which ranged from 10 to 1000 μg/mL. The final results are described as milligrams of gallic acid equivalent (GAE) per gram of dry weight (mg GAE/g dry weight).

### 2.3. Total Flavonoid Content

The total flavonoid content of the prepared methanol extract from each sample was determined using the colorimetric assay described by Kim et al. (2003) [25]. A 100-µL volume of each sample was mixed with 4 mL of distilled water in a 15-mL conical tube and 300 µL of NaNO_2_ (5%, *w/v*). The solution was incubated for 5 min; then, 300 µL of AlCl_3_ (10%, *w/v*) was added. The mixture was incubated again for 6 min; then, 2 mL of NaOH (1 M) was added. The final volume of the mixture was brought to 10 mL with deionized water. The mixture was incubated at 28 °C for 15 min, and the absorbance at 510 nm was measured. The final result was calculated using a calibration curve equivalent (standard curve equation: *y* = 0.001*x* − 0.0013, *r*^2^ = 0.9994) obtained using different concentrations of rutin (Sigma-Aldrich Co., Yongin, Korea), which ranged from 20 to 100 µg. The results are expressed as milligrams of rutin equivalent (RE) per gram of dry weight (mg RE/g dry weight).

### 2.4. Total Anthocyanin Content

The pH differential method, consisting of KCl buffer (0.025 M, pH 1.0) and CH_3_COONa buffer (0.4 M, pH 4.5), was used to determine the total anthocyanin content of the prepared methanol extract from each sample [26]. A 1-mL aliquot of the extract was mixed with 4 mL of each of the buffers and incubated at 28 °C for 15 min so that the solution could equilibrate. The absorbance reading was performed at 510 nm and 700 nm using deionized water as a blank. The absorbance (A) of each sample was calculated from an equation reported in a previous study [26]. The final result was converted to milligrams of cyanidin-3-glucoside equivalents (CGE) per gram dry weight (mg CGE/g dry weight).

### 2.5. Liquid Chromatography-Mass Spectrometry (LC-MS) Analysis for the Quantification of Phenylpropanoid Content

Phenolic compounds were identified using a system composed of an Agilent 1200 liquid chromatograph (Agilent Technologies, Palo Alto, CA, USA) coupled to a 4000 Qtrap LC/MS/MS system (Applied Biosystems Instrument, Foster City, CA, USA) in the negative ion mode ([M−H]^−^). LC-MS conditions for the *A*. *rugosa* leaf were set as follows: scan range, 100–1300 *m*/*z*; scan time, 4.80 s; curtain gas, 20.00 psi (N_2_); heating gas temperature, 550 °C; nebulizing gas, 50.00 psi; heating gas, 50.00 psi; ion spray voltage, 5500 V; declustering potential, 100 V; and entrance potential, 10 V.

### 2.6. Phenylpropanoid Extraction and HPLC Analysis

Phenylpropanoids were extracted and analyzed using a previously reported HPLC method with slight modification [27]. A total of 3 mL of aqueous methanol (80%, *v/v*) was poured into a conical tube containing 0.1 g of the freeze-dried sample powders from the different Korean mint parts. Then, it was extracted in a sonicator at 37 °C for 1 h, followed by centrifugation at 1308× *g* for 10.5 min. The supernatant was collected in a fresh tube and the whole procedure was repeated another two times. The collected aliquots were dried using nitrogen gas under a fume hood and resuspended in 3 mL of 80% methanol. An NS-4000 HPLC system (Futecs Co., Daejeon, South Korea) with a UV-Vis detector, a NS-6000 autosampler (Futecs Co., Daejeon, South Korea), and an OptimaPak C18 column (250 × 4.6 mm, 5 μm, RStech Co., Daejeon, South Korea) was used to separate the phenylpropanoids. The HPLC conditions were as follows: UV wavelength, 280 nm; flow rate, 1.0 mL/min; injection volume, 20 μL; and column temperature, 30 °C. The phenylpropanoids were separated using the gradient program previously reported by Park et al. (2017) [27]. Identification was based on the retention time and spiking experiments followed by a calculation using a corresponding calibration curve.

### 2.7. Reducing Power Assay

The reducing powers of the methanol extracts from the different organs were measured according to a previous method [28]. First, 0.2 M of phosphate buffer (pH 6.6) was prepared. Each reaction mixture contained 2.5 mL of 1% K_3_Fe(CN)_6_ and the methanol extract solutions (volumes ranged from 50 to 250 μL), to which about 2.5-mL aliquots of the buffer had been added, were then incubated at 50 °C for 20 min. The mixtures were centrifuged at 2325× *g* for 15 min after 2.5 mL of 10% trichloroacetic acid had been added. Approximately 2.5 mL of the supernatant was transferred to a fresh 15 Falcon tube and mixed with 0.5 mL of 1% FeCl_3_ and 2.5 mL of distilled water. Ascorbic acid was used as a positive control. Then, the absorbance was measured at 700 nm, and the increase in absorbance at 700 nm indicated a rise in the reducing power of each sample. All of the sample extracts were analyzed in triplicate.

### 2.8. 2,2-Diphenyl-1-picrylhydrazyl (DPPH) Free Radical Scavenging Assay

The activities of the methanol extracts from the different organs were determined according to a previous method [28]. A 0.05-mM 2,2-diphenyl-1-picrylhydrazyl (DPPH•) solution in ice-cold methanol was prepared and 200 μL of the DPPH• solution was added to each reaction mixture, which contained 3.8 mL of methanol and the extract (50–250 μL). Ascorbic acid (vitamin C) was used as a positive control in the assay. Then, the mixtures were incubated at 37 °C for 30 min in the dark, and the absorbance was measured at 517 nm. All of the sample extracts were analyzed in triplicate. The DPPH free radical scavenging activity was calculated from the equation reported in a previous study [28].

### 2.9. Hydrogen Peroxide Radical Scavenging Activity

The activities of the methanol extracts from the different organs were measured according to a previous method [24]. Briefly, a 40-mM solution of H_2_O_2_ in phosphate buffer (pH 7.4) was prepared; then, 0.6 mL of H_2_O_2_ was added to each reaction mixture, which contained 1 mL of distilled water and an aliquot of methanol extract (50–250 μL). Ascorbic acid (vitamin C) was used as a positive control. Then, the mixtures were incubated at 37 °C for 10 min, and the absorbance was measured at 560 nm. All of the sample extracts were analyzed in triplicate. The hydrogen peroxide radical scavenging activity was calculated from an equation reported in a previous study [24].

### 2.10. Superoxide Radical Scavenging Activity

The activities of the methanol extracts from the different organs were measured using nitroblue tetrazolium (NBT), according to a previously reported method [29]. Briefly, 24 mM of NBT and 1 mM of hydroxylamine hydrochloride were prepared. A total of 100 μL of NBT (24 mM) was added into the reaction mixtures, which contained 0.2 µL of 0.1 mM of ethylenediaminetetraacetic acid (EDTA) solution, 1 mL of distilled water, and the methanol extract (50–250 μL). Then, approximately 0.1 mL of hydroxylamine hydrochloride (1 mM) was added to initiate the reaction. After incubation at 25 °C for 20 min, the NBT reduction was measured at 560 nm using a reaction mixture without any extract as the control. All of the sample extracts were analyzed in triplicate. The superoxide radical scavenging activity was calculated from an equation reported in a previous study [29].

### 2.11. Extraction Process for Screening Antibacterial Activity

First, 10 g of fine powders made from each plant part was soaked in 50 mL of solvent that contained ethanol, hexane, or methanol; then, the mixture was sonicated in an ultrasound bath (JAC-4020, KODO, Technical Research Co., Ltd., Hwaseong, Korea) for 1 day. Subsequently, an extract was collected using filter paper, and then the solvent was evaporated under nitrogen gas at room temperature and stored at 4 °C until needed for the antibacterial analysis.

### 2.12. Bacterial Cultivation

Subsequently, an extract was collected using filter paper, and then the solvent was evaporated and stored at 4 °C until needed for analysis. Six bacterial strains—*Aeromonas salmonicida* (KACC 15136), *Cronobacter sakazakii* (ATCC 29544), *Escherichia coli* (KF 918342), *E*. *coli* (ATCC 35150), *Staphylococcus haemolyticus*, and *Aeromonas hydrophila* (KCTC 12487)—were obtained from the College of Medicine at Chungnam National University, Dajeon, Korea. All of the bacteria, except for *S*. *haemolyticus*, were gram-negative. The bacterial strains were shaken at 100 rpm and cultured at 37 °C to optical density at 600 nm (OD600) = 0.1 in a Luria-Bertani (LB) medium supplemented with a 50-mL aliquot of LB broth. Cultures of the mid-log phase bacteria were used for the antibacterial activity studies.

### 2.13. Screening Antibacterial Activity Using the Disc Diffusion Method

Different bacterial cultures, at an OD of 0.1, were swabbed on 30-mL LB agar plates, and five distilled discs were then placed on the agar plates [30]. Sample volumes (30 μL) of the extracts from different parts of Korean mint were added to the disc and then incubated at 37 °C overnight. An antibiotic, streptomycin (250 μg/mL), was used as the standard antibacterial agent.

### 2.14. Statistical Analysis

Analysis of variance (ANOVA) and Duncan’s multiple range tests (DMRTs) at *p* < 0.05 were performed for the data from this current study using SAS software (version 9.4, 2013; SAS Institute, Inc., Cary, NC, USA). All of the data are represented as the mean ± standard deviation of triplicate tests.

## 3. Results

### 3.1. Total Phenolic, Flavonoid, and Anthocyanin Contents

The Folin-Ciocalteu assay revealed that the greatest accumulation of total phenolics occurred in flowers (24.53 ± 1.01 mg GAE/g dry weight (dw)), followed by leaves (17.57 ± 0.91 mg GAE/g dw) and stems (7.65 ± 1.21 mg GAE/g dw). Similarly, the flowers contained the most flavonoids (18.52 ± 1.23 mg RE/g dw). This level was 1.09 times higher than that in leaves (16.91 ± 0.23 mg RE/g dw), and 2.27 times higher than that in the stems (8.17 ± 0.47 mg RE/g dw). The total anthocyanin content varied in the different parts and ranged from 0.01 to 0.22 mg CGE/g dw. The highest total anthocyanin level was detected in the flowers (0.22 ± 0.09 mg CGE/g dw), followed by the leaves (0.10 ± 0.02 mg CGE/g dw). However, the stems contained negligible amounts of anthocyanins (0.01 ± 0.00 mg CGE/g dw) (Table 1).

### 3.2. Phenylpropanoid HPLC Analysis

As shown in Table S1 and Figure S1, a total of five phenolic compounds—namely, catechin, chlorogenic acid, caffeic acid, *trans*-*p*-hydroxy cinnamic methyl ester, and ferulic acid—and three flavonoids—tilianin, rutin, and kaempferol—were identified in *A*. *rugosa* leaves by LC-MS analysis. Compounds 1 to 8 showed major ion peaks at *m*/*z* 289.5 [M−H]^−^, 445.6 [M−H]^−^, 193.6 [M−H]^−^, 353.3 [M−H]^−^, 179.4 [M−H]^−^, 609.5 [M−H]^−^, 177.7 [M−H]^−^, and 285.6 [M−H]^−^, respectively. Of these eight, six compounds (catechin, chlorogenic acid, caffeic acid, ferulic acid, rutin, and kaempferol) were quantified with HPLC. Table 2 shows the phenylpropanoid content of the flowers, leaves, and stem. The levels of these phenylpropanoids varied among the three parts. The total phenylpropanoid content ranged from 0.433 to 1.570 mg/g dw), and the highest level was recorded in leaves. Among the individual phenylpropanoids, the highest levels of catechin, caffeic acid, and ferulic acid were obtained from leaves, and these levels were 37.101-fold, 1.202-fold, and 1.528-fold higher, respectively, than the lowest levels obtained from stems. Ferulic acid and kaempferol were not detected in the stems, but they did contain the most chlorogenic acid.

### 3.3. In Vitro Antibacterial Activity

**Figure 1:**
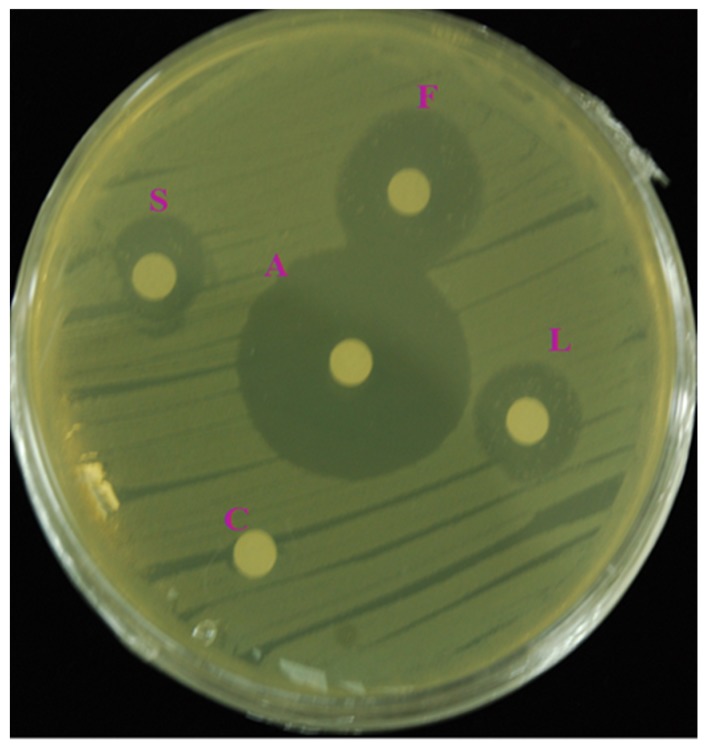
Representative image showing antibacterial activity against a bacterial pathogen. A—antibiotic (Streptomycin); C—control (methanol); F, L, and S are methanolic extracts; F—flower extract, L—leaf extract, and S—stem extract.

The in vitro antibacterial activity tests revealed a zone of inhibition around the edge of the disc for each extract (Figure 1). The inhibition zones generated by the methanol extracts from the various plant parts are shown in Table 3. The methanolic extracts showed the most promising activities against the six bacterial strains (these results are not shown). Therefore, the ethanolic and hexane extracts were excluded from the study, and the methanolic extracts were used in subsequent studies. The methanol extract from the flowers had greater antibacterial properties than the stem and leaf extracts. In particular, the flower extracts possessed the greatest antibacterial activity against *E*. *coli* (KF 918342), followed by *A*. *hydrophila*, *S*. *haemolyticus*, *A*. *salmonicida*, *E*. *coli* (ATCC 35150), and *C*. *sakazakii*, in that order. The stem extracts had the lowest antibacterial activities against all the bacterial strains tested.

### 3.4. In Vitro Antioxidant Assays

**Figure 2:**
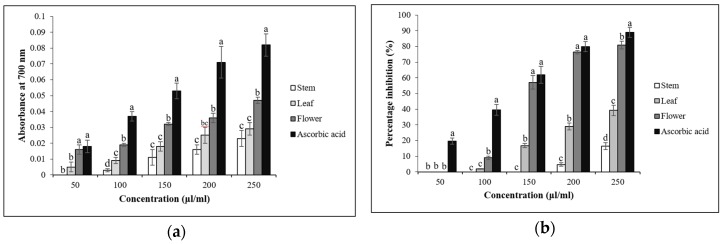

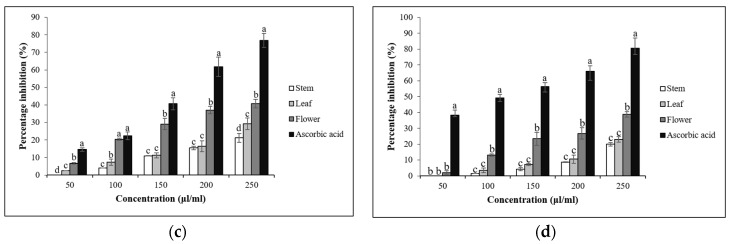
Antioxidant activity of the methanol extracts of stem, leaves, and flowers of *A*. *rugosa*. (**a**) Reducing power assay; (**b**) DPPH radical scavenging assay; (**c**) Hydrogen peroxide radical scavenging assay; (**d**) Superoxide radical scavenging assay. All of the values in the figure are expressed as means (%) of triplicated experiments and standard deviation of three experiments. Mean values with a different letters (a, b, c, and d, respectively) were significantly different (*p* < 0.05, ANOVA, DMRT).

The reducing power of the extracts increased in a concentration-dependent manner. In particular, the flower extracts had the highest antioxidant capacity among the different parts tested (Figure 2a). The DPPH free radical scavenging activity was determined using methanol extracts from the different parts of Korean mint at different concentrations (50 to 250 μg/mL; Figure 2b). The flower extracts had the highest antioxidant capacity among the parts tested. Methanol extracts from the flowers, leaves, and stems had 80.9 ± 1.1%, 39.2 ± 1.2%, and 16.3 ± 0.7% DPPH activities, respectively, at a concentration of 250 μL/mL, whereas the ascorbic acid DPPH activity was 88.9 ± 0.8%. The hydrogen peroxide radical scavenging activity of the extracts from the different plant parts was also concentration-dependent (Figure 2c). This assay showed that an increase in the concentration of the extracts led to a rise in the radical scavenging activity. At 250 μL/mL, the flower extract had the highest scavenging activity (40.8 ± 0.8%), whereas the radical scavenging activity of ascorbic acid was 76.8 ± 0.6%. Superoxide radical scavenging activity was determined using methanol extracts at different concentrations (50 to 250 μg/mL; Figure 2d). At 250 μL/mL, ascorbic acid had 80.6 ± 0.3% scavenging activity, whereas the scavenging activities of the methanol extracts from the flowers, leaves, and stems were 38.8 ± 1.1%, 23 ± 1.4%, and 20 ± 0.6%, respectively.

## 4. Discussion

In this study, the methanol extracts from the different parts had wide-spectrum antibacterial activity. However, the flower extract had a greater efficacy than the leaf and stem extracts. The antioxidant activities of the methanol extracts from the different plant parts were measured as the ability of the extracts to scavenge hydroxyl radicals. The methanol extracts had concentration-dependent antioxidant properties. The flower extracts had the greatest potential antioxidant activity, which was possibly because they had higher phenylpropanoids levels. Korkina (2007) reported that phenylpropanoids, the largest group of plant secondary metabolites, are considered to be naturally occurring antioxidant and antibacterial agents [31].

Three flavonoids (kaempferol, catechin, and rutin) and three phenolic acids (ferulic acid, chlorogenic acid, and caffeic acid) were identified in the three different *A*. *rugosa* parts after the HPLC analysis. These findings were in agreement with previous studies, which had also reported the presence of ferulic acid in *A*. *rugosa* cells [32], chlorogenic acid and caffeic acid in *A*. *rugosa* [33,34], and quercetin and kaempferol in *Agastache mexicana* [35]. Phenolic compounds are natural antioxidants that can donate electrons to oxidative molecules [36,37]. Catechin, rutin, and kaempferol have been reported to possess effective antioxidant properties [38,39]. Anthocyanins, which belong to the flavonoid class, are also powerful natural antioxidants [40]. Hydroxybenzoic acid (4-hydroxybenzoic acid) and hydrocinnamic acids (chlorogenic acid, ferulic acid, and caffeic acid) are considered to be potential antioxidants [41,42]. Furthermore, the phenolics identified in this study have been reported to possess effective antibacterial properties [43,44,45,46,47].

The in vitro antioxidant and antibacterial activity assays showed that antibacterial and antioxidant activities were higher in the flowers than in the stems and leaves. These properties might be due to the higher phenolic levels in flowers. A previous study reported that flowers produced the most rosmarinic acid, tilianin, and acacetin, which possess antibacterial and antioxidant activities [11]. Furthermore, this study showed that flowers had the highest total flavonoid, anthocyanin, and phenolic contents, even when the leaves contained higher levels of ferulic acid, caffeic acid, and catechin. Therefore, the increased accumulation of phenylpropanoid compounds in flowers explains their greater antibacterial and antioxidant activities compared to stems and leaves. The findings are consistent with previous studies, which demonstrated that red cultivars of *Lactuca sativa* had higher concentrations of total anthocyanins, flavonoids, and phenolics, and thus showed higher antioxidant activities than green cultivars [48]. Furthermore, the red cultivar of *Raphanus sativus* contained higher levels of phenolic compounds and exhibited greater antioxidant effects than the white cultivar did [26].

All of the plant parts, including the wood, seed, bark, stem, pod, leaf, fruit, root, flower, and pollen, contain diverse natural antioxidants [49]. In particular, flowers are considered to be a potential source of bioactive phytochemicals with high antioxidant [50]. In this study, the flowers showed higher antioxidant ability than the stems and leaves. This finding was consistent with a previous study describing that the extract of the *Ferulago angulate* flower indicated better activity in DPPH radical scavenging than that of leaf and stem extract [51]. Bettaieb et al. (2010) also reported that the essential oil and acetone extract of *Cuminum cyminum* L. flowers were found to be the most effective in the DPPH, β-carotene/linoleic acid, and reducing power assays compared to those of the stem and leaves [52]. Furthermore, the flowers of *Ixora coccinea* possessed higher antioxidant ability than the stem and leaves [53].

The methanol extracts from the different parts of *A*. *rugosa* possessed antibacterial activities against all the bacterial strains tested. They also had antioxidant properties. However, the plant parts had significantly different antibacterial and antioxidant activities. These differences may be due to the different phytochemical constituents of the extracts. The secondary metabolites in the different parts of Korean mint have differing effects, including antioxidant and antibacterial activities [54,55] *Agastache* species have attracted great attention because these plants contain several phytochemicals and can be used as seasoning agents, flavoring agents, spices, herbs, and functional foods. They have also been exploited by the medical and cosmetic industries as antimicrobial and antioxidant agents.

Variations in the results obtained for the stems, leaves, and flowers of Korean mint or potential variations between the results reported by previous studies and those reported by this study are not surprising. This may be because ecological factors have led to variations in the chemical composition [56], and/or differences in the extraction solvents, seasons, and the phytochemicals that are present [57]. Furthermore, environmental factors, including drought or excessive rainfall, can affect the quantity and quality of bioactive compounds [58].

The World Health Organization (WHO) has reported that about 80% of the global population relies on herbal remedies for their primary healthcare [59,60]. This study showed that the different parts of *A*. *rugosa* have different antibacterial and antioxidant activities. These properties could be successfully used to treat several diseases caused by oxidative stress and bacterial infections. This may indicate that the medicinal plants exploited in traditional remedies may possess beneficial biological activities, including antioxidant or antimicrobial activities. Substantial research is being conducted by pharmaceutical and biomedical corporations into traditional remedies [61,62,63] and the toxicology of the phytomedical compounds in such preparations [64,65]. Therefore, this study supports the potential use of *A*. *rugosa* stems, leaves, and flowers in traditional herbal medicine applications.

## 5. Conclusions

Phenolic compounds were quantified in the leaves, flowers, and stems of *A*. *rugosa* by HPLC analysis, and the total anthocyanin, flavonoid, and phenolic contents were assessed. The methanol extracts of the different plant parts were found to have significant antibacterial and antioxidant activities. The flowers contained the highest total phenolic, flavonoid, and anthocyanin levels, which explains why they had greater antibacterial and antioxidant effects than the leaves and stems. This study shows that there are synergistic antimicrobial and antioxidant capabilities that are derived from phenylpropanoids in the stems, leaves, and flowers of *A*. *rugosa*.

## Figures and Tables

**Table 1:** Total phenolics, flavonoid, and anthocyanin of the stem, leaves, and flowers of *A*. *rugosa.*

Organ	Total Phenolics (mg GAE/g dw)	Total Flavonoid (mg RE/g dw)	Total Anthocynin (mg CGE/g dw)
Flower	24.53 ± 1.01 ^a^	18.52 ± 1.23 ^a^	0.22 ± 0.09 ^a^
Leaf	17.57 ± 0.91 ^b^	16.91 ± 0.23 ^b^	0.10 ± 0.02 ^b^
Stem	7.65 ± 1.21 ^c^	8.17 ± 0.47 ^c^	0.01 ± 0.00 ^b^

Mean values with a different letters (a, b, and c, respectively) were significantly different (*p* < 0.05, ANOVA, DMRT) in the columns. DMRT: Duncan’s multiple range test, GAE/g dw: gallic acid equivalent per gram of dry weight; RE/g dw: rutin equivalent per gram of dry weight; CGE/g dw: cyanidin-3-glucoside equivalents per gram dry weight.

**Table 2:** The accumulation of phenolic compounds in the stem, leaves, and flowers of *A*. *rugosa.*

Organ	Phenolic Compounds (mg/g dw)						
	Catechin	Chlorogenic Acid	Caffeic Acid	Ferulic Acid	Rutin	Kaempferol	Total
Flower	0.014 ± 0.005 ^b^	0.011 ± 0.006 ^b^	0.267 ± 0.001 ^b^	0.815 ± 0.001 ^b^	0.061 ± 0.009 ^ab^	0.137 ± 0.007 ^a^	1.305 ± 0.024 ^b^
Leaf	0.148 ± 0.005 ^a^	0.027 ± 0.021 ^b^	0.327 ± 0.030 ^a^	0.830 ± 0.001 ^a^	0.081 ± 0.012 ^a^	0.157 ± 0.021 ^a^	1.570 ± 0.064 ^a^
Stem	0.004 ± 0.003 ^c^	0.103 ± 0.010 ^a^	0.272 ± 0.003 ^b^	ND	0.053 ± 0.013 ^b^	ND	0.433 ± 0.022 ^c^

Mean values with a different letters (a, b, and c, respectively) were significantly different (*p* < 0.05, ANOVA, DMRT) in the columns. ND: not detected.

**Table 3:** Antibacterial activity of the methanol extracts of stem, leaves, and flowers, of *A*. *rugosa.*

Bacterial Strain	Zone of Inhibition (mm)			
	Flower	Leaf	Stem	Streptomycin
*E.coli* (KF918342)	18.3 ± 0.5 ^b^	14.0 ± 1.0 ^c^	11.0 ± 1.0 ^d^	27.6 ± 0.6 ^a^
*Staphylococcus hemolyticus*	16.6 ± 0.5 ^b^	12.0 ± 1.0 ^c^	8.0 ± 1.0 ^d^	26.3 ± 0.6 ^a^
*Aeromonas hydrophila*	17.0 ± 1.0 ^b^	14.6 ± 0.5 ^c^	7.6 ± 0.5 ^d^	27.0 ± 0.0 ^a^
*E.coli* (ATCC 35150)	16.3 ± 1.5 ^b^	13.6 ± 1.5 ^c^	10.0 ± 1.0 ^d^	28.3 ± 0.6 ^a^
*Cronobacter sakazakii*	15.0 ± 1.0 ^b^	12.0 ± 1.7 ^c^	9.3 ± 0.5 ^d^	25.6 ± 0.6 ^a^
*Aeromonas salmonicida*	16.6 ± 0.5 ^b^	9.3 ± 0.5 ^c^	8.0 ± 1.0 ^c^	27.0 ± 1.0 ^a^

Mean values with a different letters (a, b, c, and d, respectively) were significantly different (*p* < 0.05, ANOVA, DMRT) in the columns.

## Supplementary Materials

The following are available online at https://www.mdpi.com/2076-3921/8/3/75/s1, Figure S1: LC-MS spectrum of phenolic compounds identified in leaves of *A*. *rugosa*. 1, Catechin; 2, Tilianin; 3, Ferulic acid; 4, Chlorogenic acid; 5, Caffeic acid; 6, Rutin; 7, *trans*-p-hydroxy cinnamic methyl ester; 8, Kaempferol., Table S1: List of detected phenolic compounds and their retention times (t*_R_*), and molecular ([M−H]^–^) and fragment ions in negative mode in *A*. *rugosa* leaf.
