# Supplementary material for: In Vitro Antioxidant and Antimicrobial Properties of Flower, Leaf, and Stem Extracts of Korean Mint
Source: Antioxidants (Basel). 2019 Mar 26;8(3):75. doi: 10.3390/antiox8030075 (PMC6466538; doi:10.3390/antiox8030075)
Supplement: Supplementary file 1 [file antioxidants-08-00075-s001.pdf]

Table S1. List of detected phenolic compounds and their retention times (t_R), and molecular ($[M–H]^-$) and fragment ions in negative mode in *A. rugosa* leaf

No	Name	Molecular Formula	Molecular Weight	t_R (min)	$[M – H]^-$
1	Catechin	$C_{15}H_{14}O_6$	290.27	20.4	289.5
2	Tilianin	$C_{22}H_{22}O_{10}$	446.41	21.9	445.6
3	Ferulic acid	$C_{10}H_{10}O_4$	194.18	27.5	193.6
4	Chlorogenic acid	$C_{16}H_{18}O_9$	354.31	32.5	353.3
5	Caffeic acid	$C_9H_8O_4$	180.16	35.8	179.4
6	Rutin	$C_{27}H_{30}O_{16}$	610.52	77.9	609.5
7	*Trans*-*p*-hydroxy cinnamic methyl ester	$C_{10}H_{10}O_3$	178.18	31.6	177.7
8	Kaempferol	$C_{15}H_{10}O_6$	286.23	82.2	285.6

Figure S1. LC-MS spectrum of phenolic compounds identified in leaves of A. rugosa. 1, Catechin,; 2, Tilianin; 3, Ferulic acid; 4, Chlorogenic acid; 5, Caffeic acid; 6, Rutin; 7, trans-p-hydroxy cinnamic methyl ester; 8, Kaempferol.

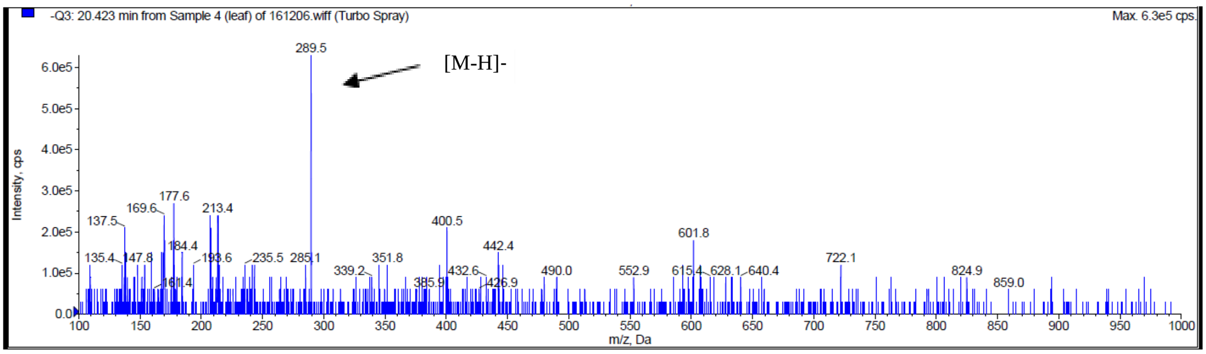

1. Catechin (MW.290.27)

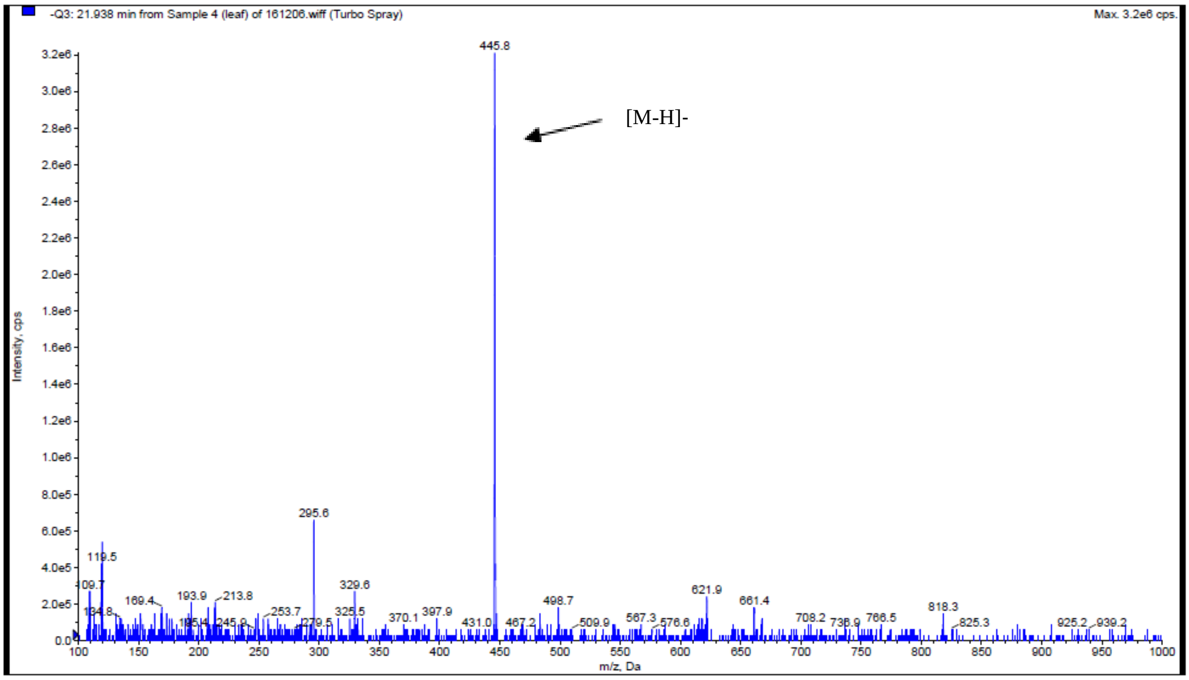

2. Tillianin (MW.446.41)

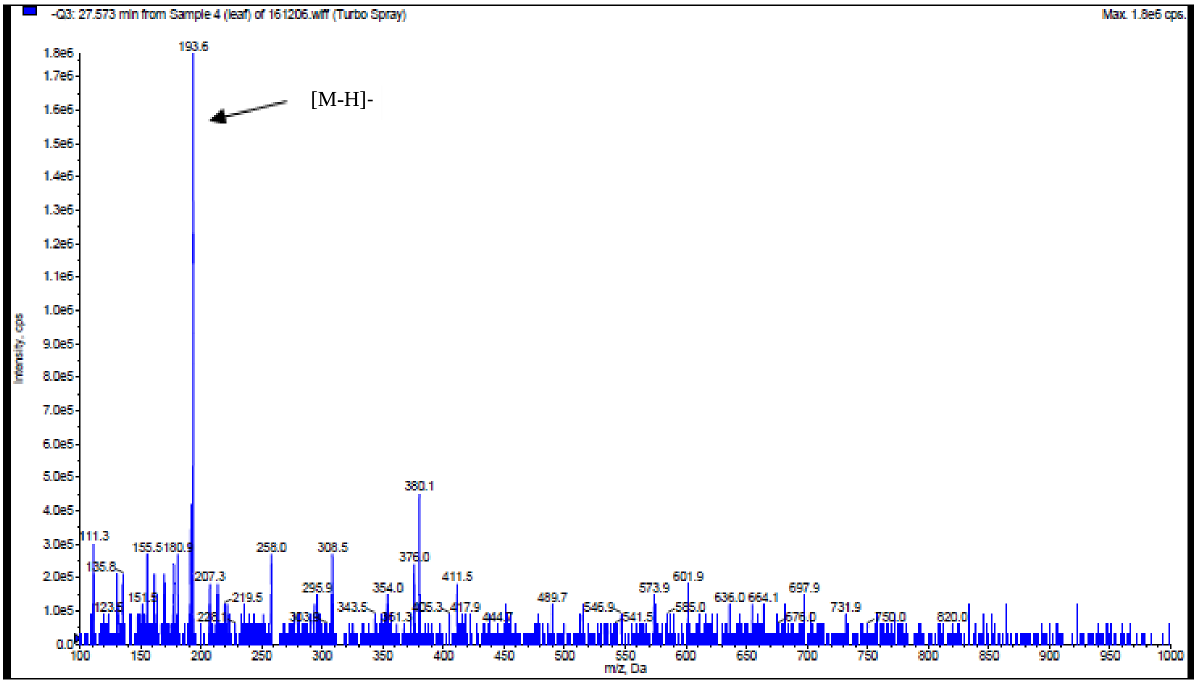

3. Ferulic acid (MW.194.18)

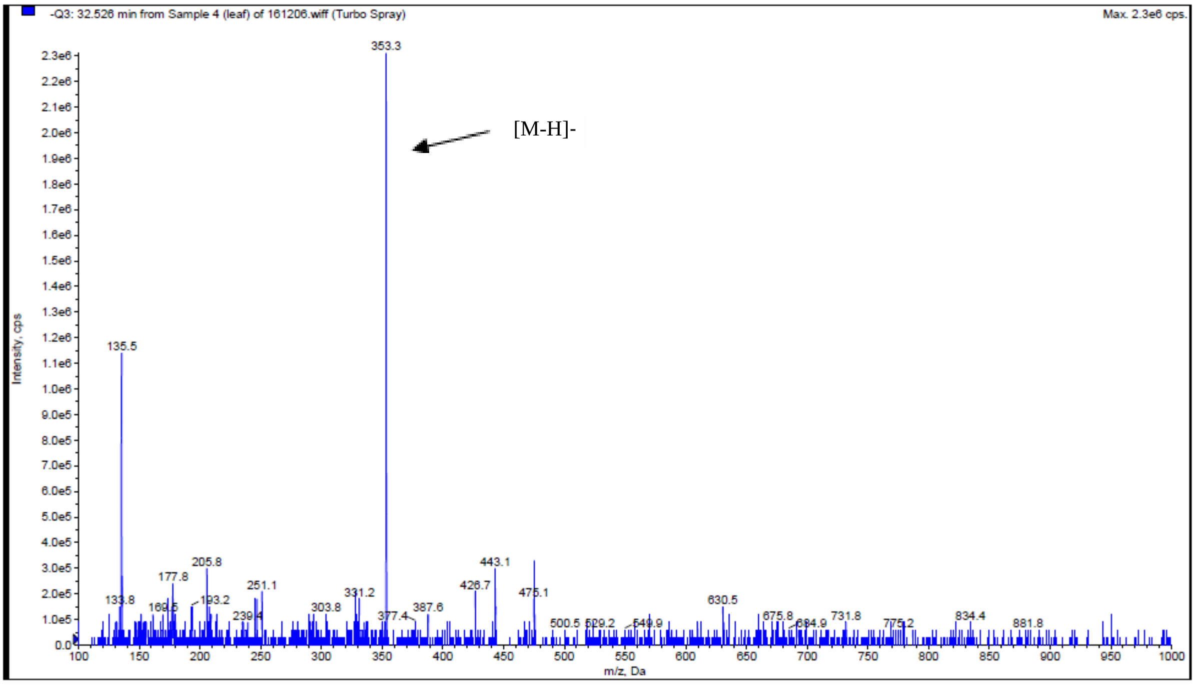

4. Chlorogenic acid (MW.354.31)

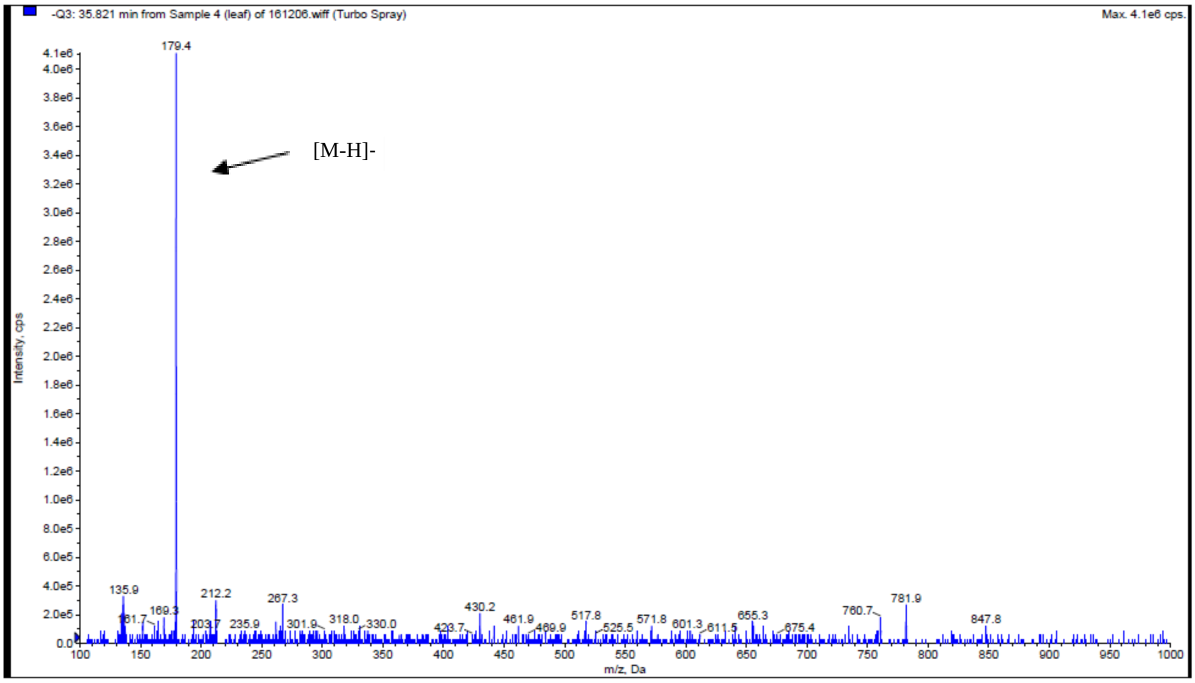

5. Caffeic acid (MW.180.16)

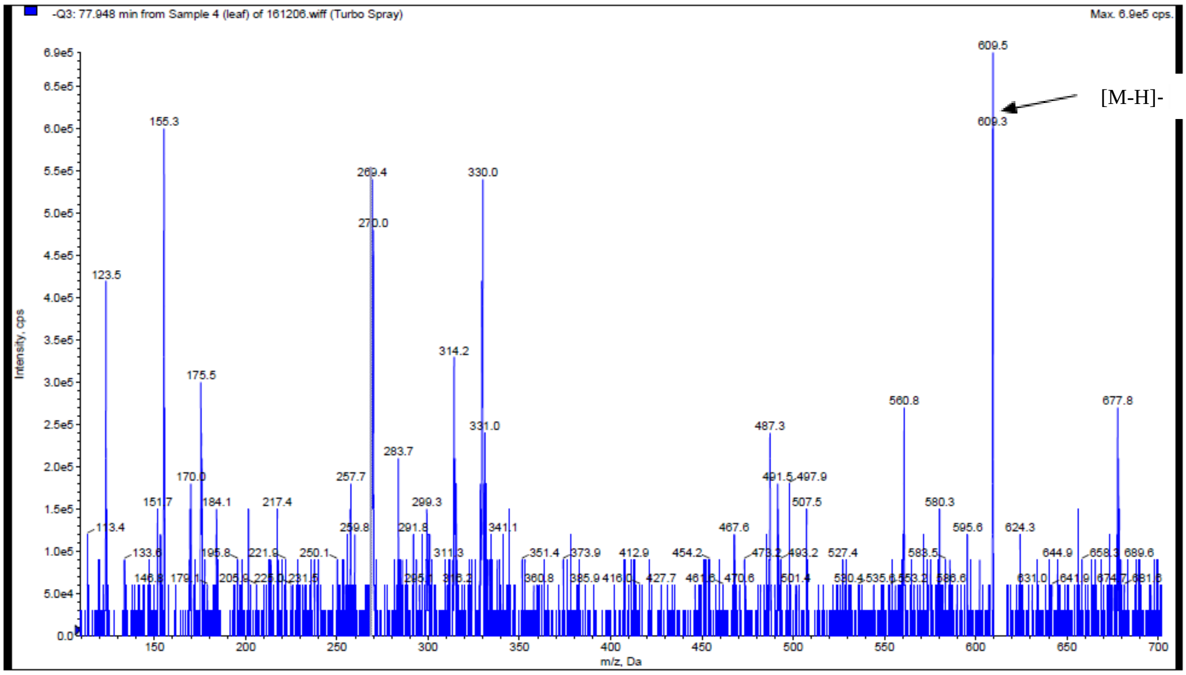

6. Rutin (MW.610.52)

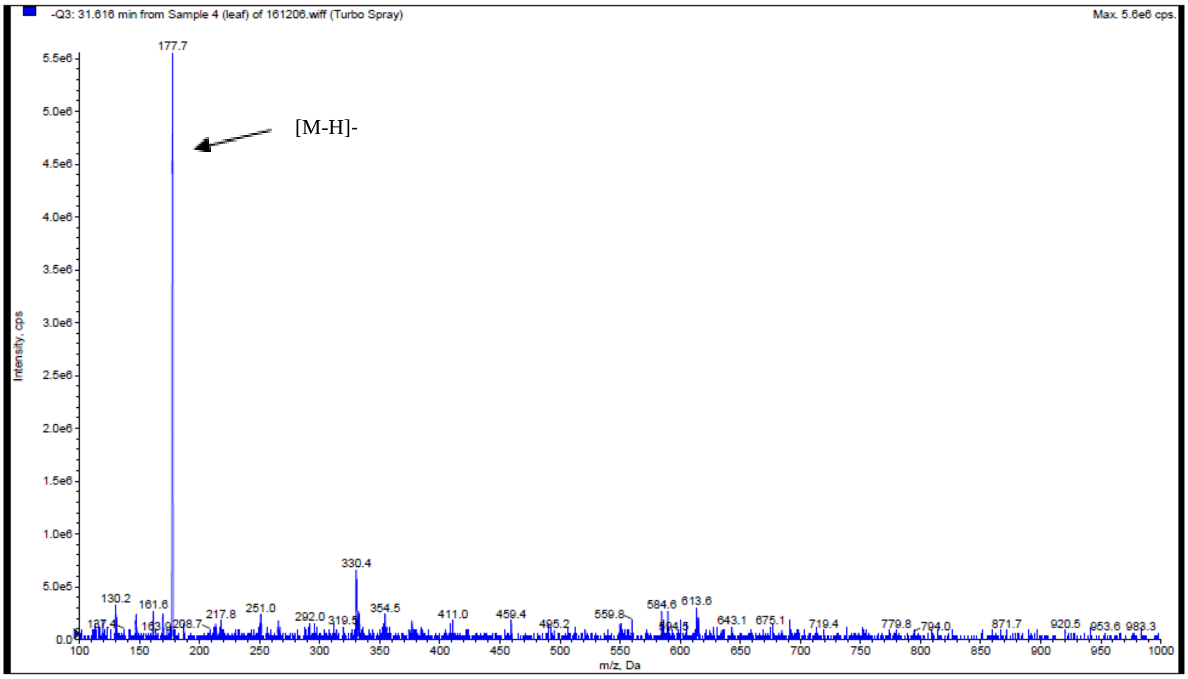

7. *trans*-p-hydroxy cinnamic methyl ester (MW.178.18)

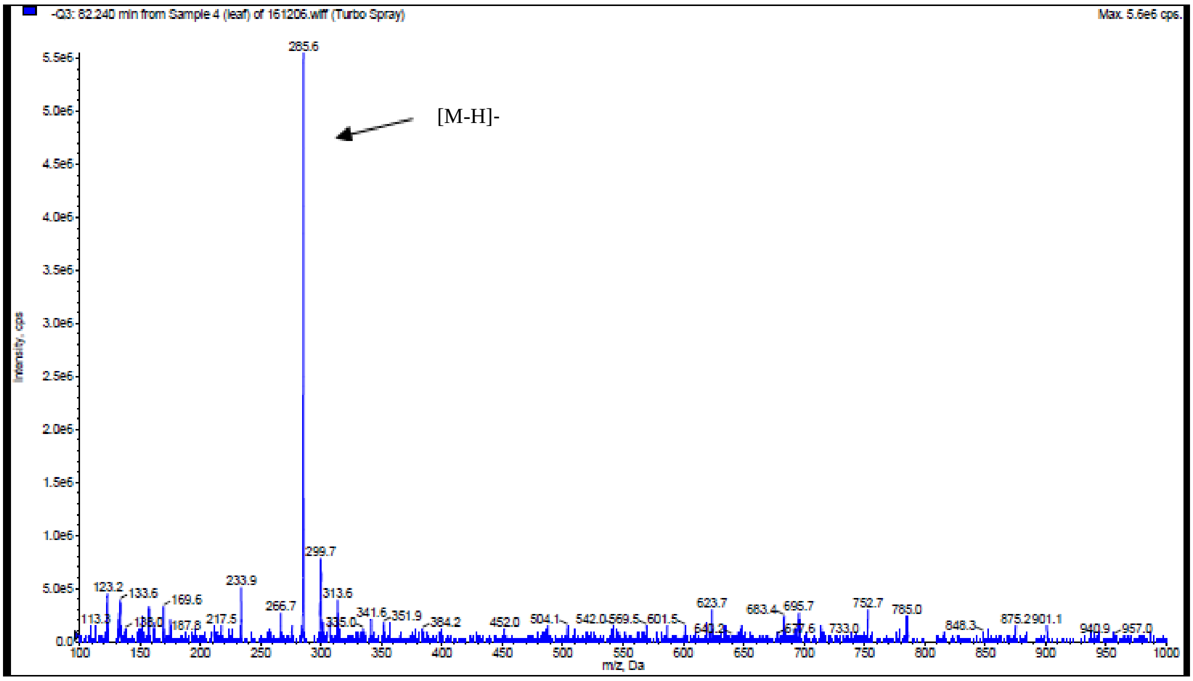

8. Kaempferol (MW.286.23)
